# Pulmonary Eosinophilic Inflammatory Infiltration Post-Intensive Care in a Nearly Drowned Young Man with Papillary Fibroelastoma: A Rare Complication Discovered by Forensic Autopsy

**DOI:** 10.3389/fmed.2017.00253

**Published:** 2018-01-15

**Authors:** Gelsomina Mansueto, Emanuele Capasso, Claudio Buccelli, Massimo Niola

**Affiliations:** ^1^Department of Advanced Biomedical Sciences-Pathology Section, University of Naples Federico II, Napoli, Italy; ^2^Legal Medicine Section, University of Naples Federico II, Napoli, Italy

**Keywords:** papillary fibroelastoma, eosinophilic inflammatory infiltration, nearly drowned, intensive care, syncope

## Abstract

Papillary fibroelastoma is a rare benign lesion of heart (1). It is the second most common primary cardiac neoplasm, accounting for 4.4–8% of all tumors of the heart (2). We described a forensic autopsy of a nearly drowned young man with cardiac papillary fibroelastoma who died because of a pulmonary inflammatory infiltration rich in granulocytes after intensive care. This occurrence is rare but possible and should be kept in mind because a lung inflammatory infiltrate rich in eosinophilic granulocytes can be present in different pathological conditions and differential diagnoses are often difficult to do.

## Introduction

**Figure 1:**
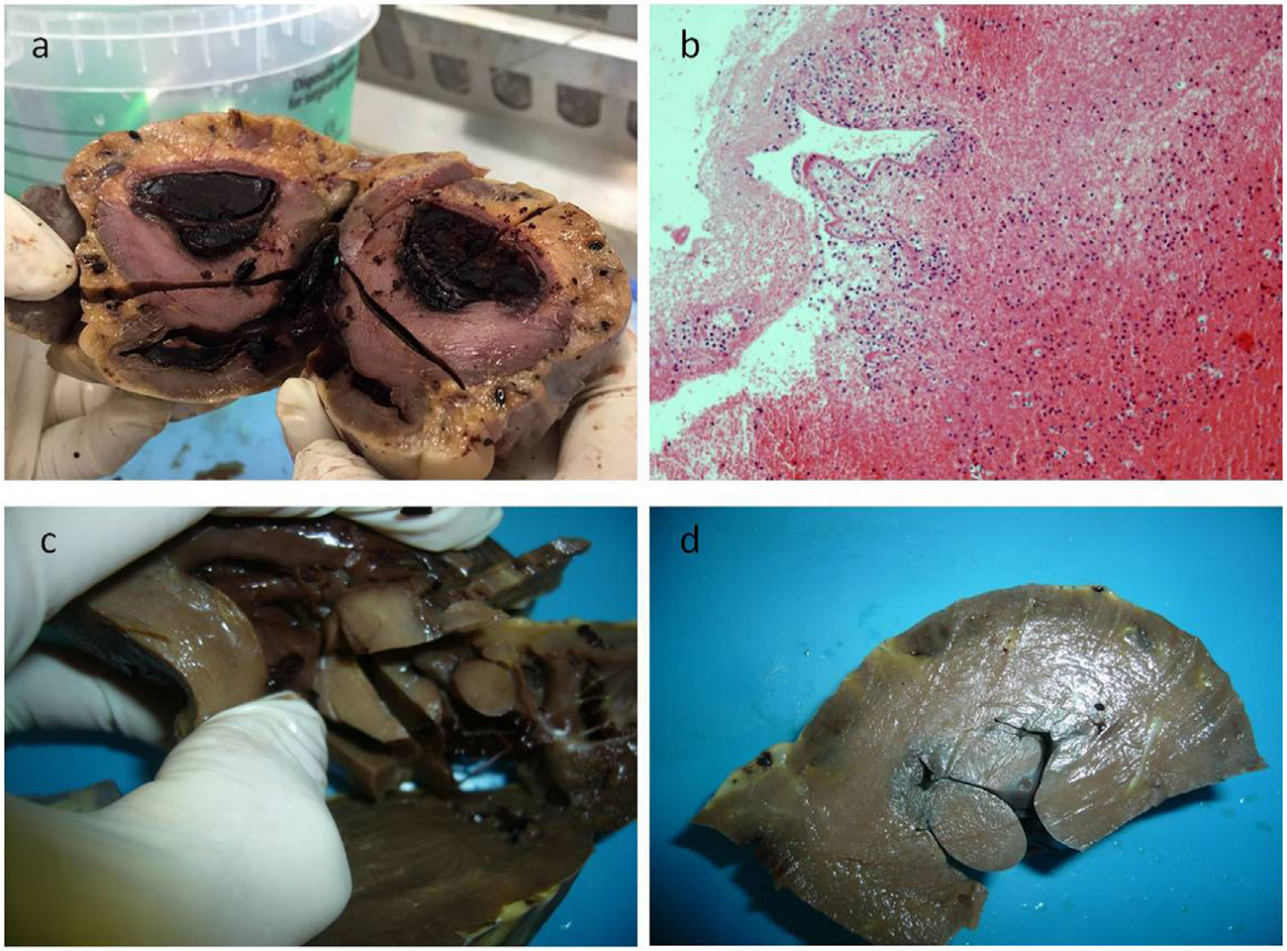
In panel **(A)**, the gross appearance of the lesion, and in panel **(B)** the microscopic of the blood clot that was first found during dissecting the heart, consisting in fibrin and poor in cells. After removing the clot, a papillary multiple lesion was found, the base of which was in the wall of the ventricle **(C,D)**.

**Figure 2:**
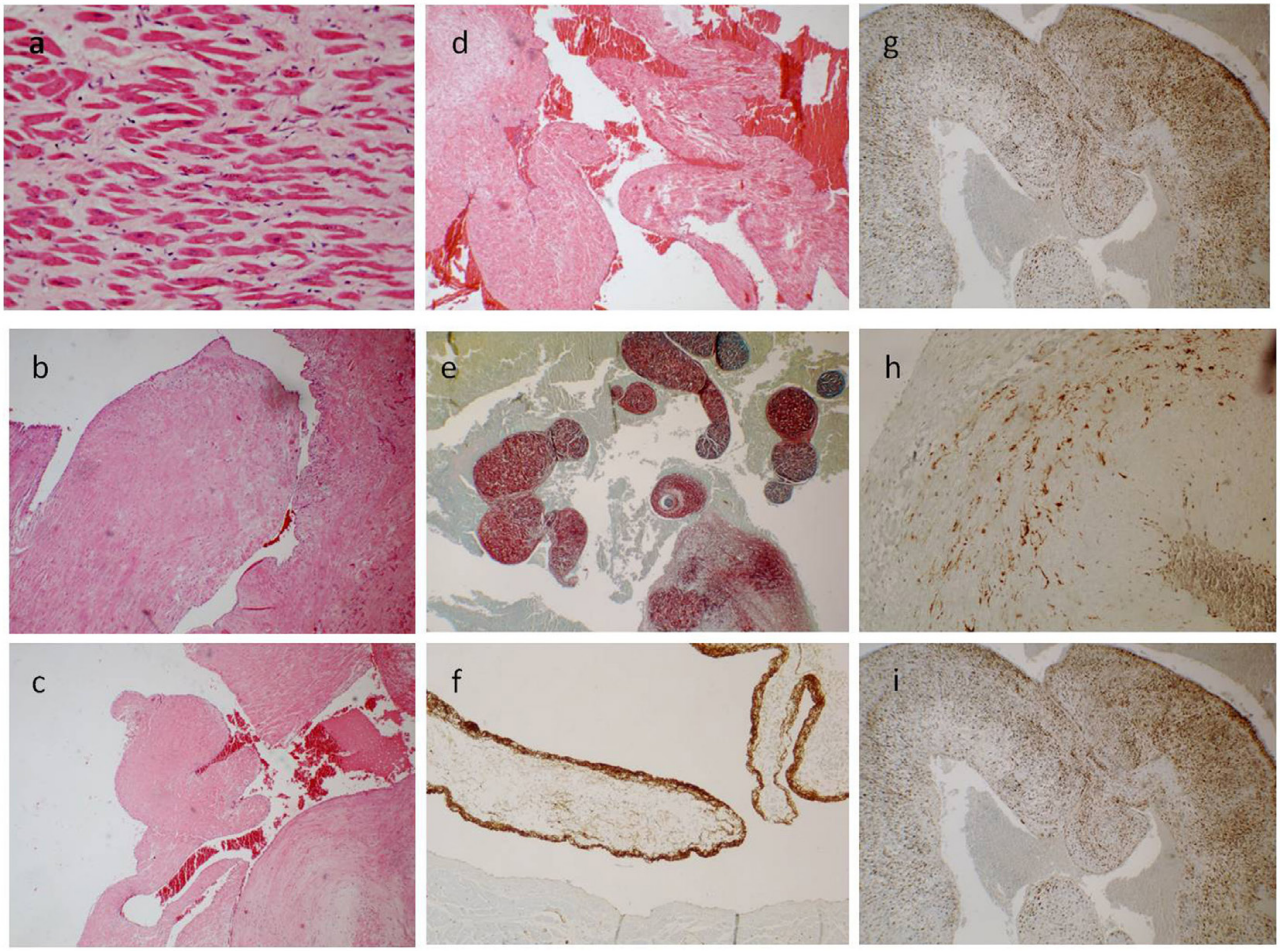
**(A)** The ventricle hypertrophy (E/E 63×). **(B–D)** The papillary ventricular lesion consisted mainly in connective tissue, poor in cells [E/E 20× in **(B)**, 10× in **(C,D)**]. **(E)** Collagen and elastic fibers (special stain. Sirius Red 10×). **(F)** The surface was positive for anti-FVIII (40×); and a diffuse, sparse positivity for anti-CD34 [20× in **(G)**] and anti-S-100 superficial and spars [20× in **(I)**] was found; few cells instead were positive for anti-Actin [40× in **(H)**].

**Figure 3:**
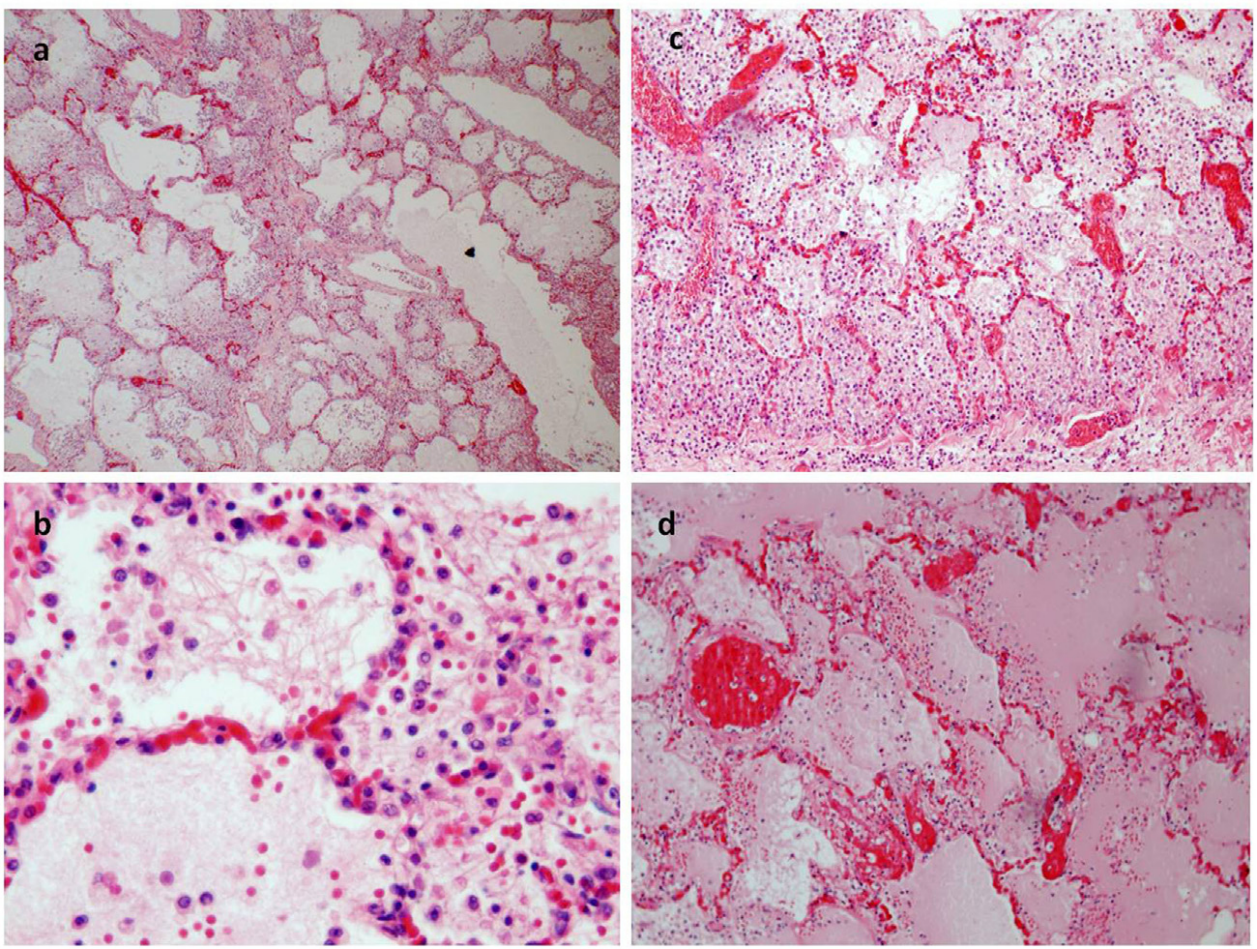
**(A)** Pulmonary emphysema (E/E 20×). **(B–D)** The alveoli were filled an effusion rich in protein and a rich infiltrate of eosinophil granulocytes too [E/E 63× in **(B)**; 40× in **(C)**]. **(D)** The congestion of pulmonary vessels and proteinaceus aedema (E/E 40×).

**Figure 4:**
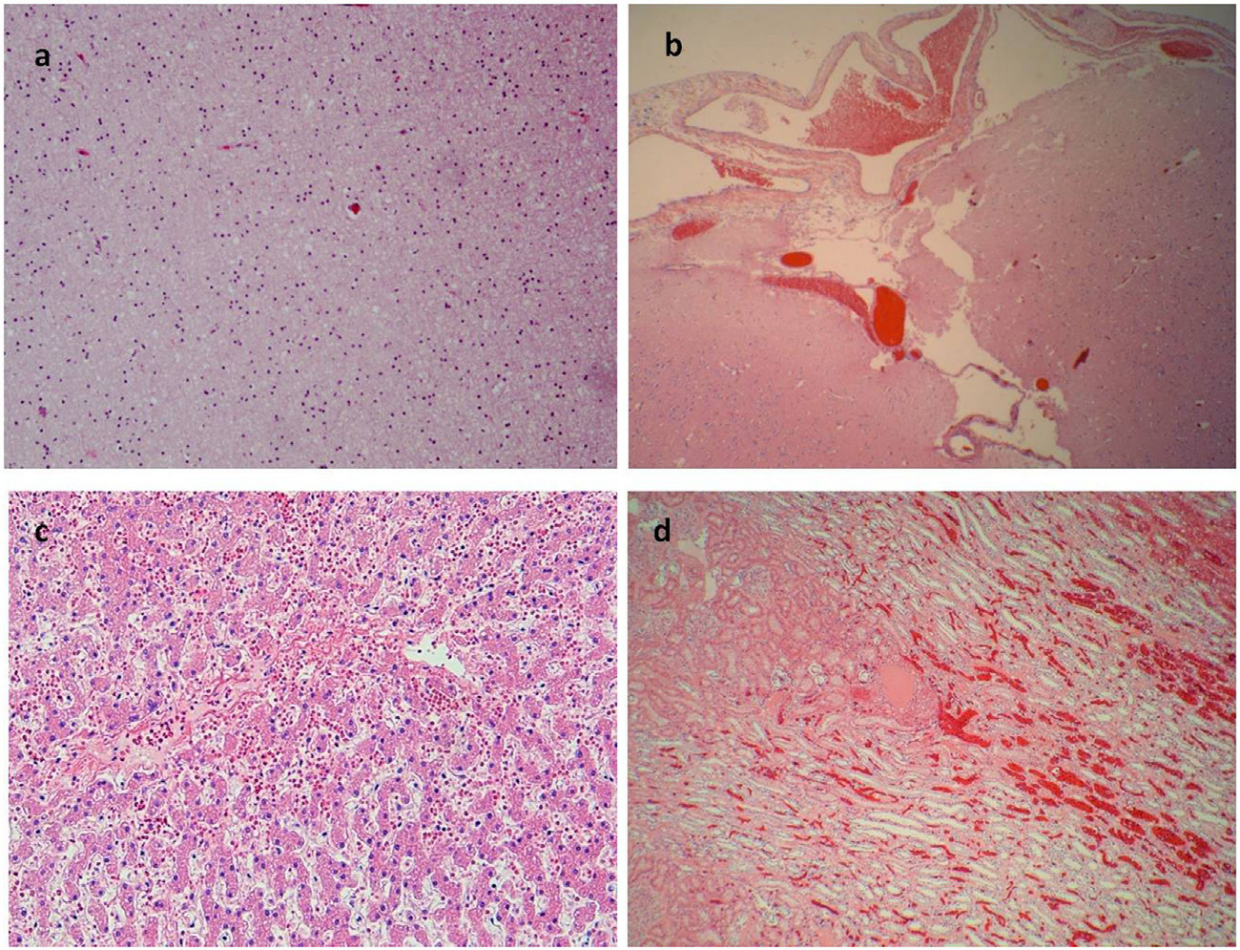
More signs of asphyxia. **(A)** Brain edema (E/E 40×). **(B)** Congestion of subdural blood vessels (E/E 10×). **(C)** Edema and erythrocytes in the liver sinusoid (E/E 40×). **(D)** Congestion of kidney vessels (E/E 20×).

A 17-year-old man was sent in intensive care because of drowning. Cranium, chest, and abdomen CT was negative. The hemogasanalysis showed hypercapnia and respiratory acidosis. Therefore, he underwent mechanical ventilation, but in spite of this, his acidosis grew worse, and he was intubated. The patient’s situation worsened a day after, because of the onset of hypotension (PA = 79/40 mmHg), tachyarrhythmia (HR = 128 bpm), and PaO_2_ < 79%. He died about 36 h after admission. Forensic autopsy was requested to identify the time, cause, and means of the death. Toxicology was negative for all drugs. Autopsy showed focal sub-meningeal hemorrhage and outer inspection of neck, chest, and abdomen instead was negative. By opening the thorax, the surface of lungs was congested and sub-pleural spots were recognized as Tardieu’s spots, a well-known sign of asphyxia. The silhouette of heart was preserved, and the coronary vessels were not occluded; but the thickness of the ventricles was increased, both in the left one (2.3 cm; normal 1.3–1.5 cm) and right one (1.3 cm; normal 0.3–0.5 cm), and in the septum (1 cm) and in their lumen a big blood clot was found (Figures 1A,B). After removing the clot, we found a papillary, whitish, multifocal lesion, the base of which was in the wall of the ventricle (Figures 1C,D). Histologically, in both the ventricles and the interventricular sept the number of muscle cells was increased, with an interposition of fibrous tissue confirming the cardiac hypertrophy (Figure 2A). Samples of the multiple papillary lesions were taken, and the hematoxylin and eosin stain (H&E) showed an extracellular fibrous matrix with elastic component and few cells interposed, while a single layer of cells covered the structure (Figures 2B–D). We performed histochemical special stains that confirmed the presence of mainly collagen and elastic fibers (Figure 2E). Immunohistochemistry showed a positivity for anti-CD34 and anti-S-100 in the whole lesion, positivity for anti-FVIII on the surface and focal positivity for actin (Figures 2F–I). This morphology and immunophenotype were compatible with a cardiac papillary fibroelastoma. Histology of the lungs showed an irregular emphysema (Figure 3A), congested vessels, and the hemorrhagic spots in pleura, typical aspects of asphyxia and hypoxia. Furthermore, the alveoli were filled by proteinaceous exudates with eosinophilic granulocytes and erythrocyte extravasation (Figures 3B–D). Moreover, brain, liver, and multiorgan edema, congestion, and erhitrocyte extravasation were detected (Figures 4A–D). Our final diagnosis was of a death by eosinophilic pneumonia post-intensive care in a nearly drowned young man affected by cardiac papillary fibroelastoma.

## Discussion

Papillary fibroelastoma, also called fibropapilloma, or fibroelastic hamartoma, or Lambl’s excrescence, is a rare benign lesion of heart (1). It is the second most common primary cardiac neoplasm, accounting for 4.4–8% of all tumors of the heart (2). The incidence is relatively low, but statistic dates are actually quite different from hospital to hospital. It is usually found accidentally in patients, either during magnetic resonance imaging, echocardiography, during cardiac surgery or autopsy (3, 4). The lesion can be seen at any age (mean: 60 years). Papillary fibroelastomas can be either single or multiple, the latter possibility especially if it develops on a damaged tissue, with valvular or ventricular or atrium location (5, 6). Clinically, the biggest lesions cause an obstruction of cardiac valves, where the lesion usually develops, or take a variable space in the atria or ventricles, so that symptoms that we can found are mainly related to defects of ejection of blood, i.e., hypertension in the pulmonary circle (for fibropapillomas that occur in the left sections of heart) or the systemic one (if they develop in the right sections). Smaller lesions can be asymptomatic even during the whole of patient’s life and be found only accidentally during an imaging test or an autopsy, but they can easily break up with subsequent embolization. The consequences, therefore, can be extremely variable, from mesenteric angina to transient ischemic attacks, from claudicatio intermittens of limbs to arrhythmias and syncope or stroke. Therefore, irrespective of the size, papillary fibroelastoma can have a severe prognosis quoad valetudinem or quoad vitam (7). Histologically the papillary neoplasm is composed of variably thickened, broad papillae of varying lengths, lined by endothelial cells, with avascular connective tissue stroma, with variable, myxoid, fibrosis, or collagen (5). Immunohistochemical markers included, collagen type IV, muscle-specific actin, desmin, factor VIII-related antigen, CD34, S-100 protein, and calretinine. The cells covering the surface are positive for factor VIII-related antigen and CD34, in keeping with their presumed vascular endothelial origin, but also the S-100 protein may be positive (8).

In our case, we formulated the hypothesis of an arrhythmia, consequent to ventricles impairment, that caused a syncope and an accidental nearly drowning. Once rescued, the young man was admitted to hospital. He died after the intensive care, 36 h after the accident. Histology of the lungs, as described previously, showed irregular emphysema, congested vessels, hemorrhagic aspects, and a proteinaceous edema with eosinophilic granulocytes. The presence of a pulmonary emphysema was compatible with a death by drowning and, therefore, by asphyxia, because in the first phase of nearly all the asphyctic conditions, one of the main mechanisms is a series of contractions of diaphragm, that let intrathoracic pressure increase, thus rupturing the alveolar septa and causing emphysema. The marked vascular congestion and extravasation of erythrocytes are compatible with asphyxia and hypoxia. In this case, a special feature consisted in pulmonary eosinophilia. Pulmonary eosinophilia is a rare condition but its diagnosis is challenging, because it can have the same diverse clinical and radiographic presentations seen in other common pulmonary conditions. Radiological manifestations show pulmonary infiltrates, characterized by foci of air-space consolidation and focal ground-glass opacities, can be seen in pulmonary eosinophilia of all causes (9). Clinicians should be alert to these syndromes and must think of them in any lung disease differential diagnoses. In fact, apart idiopathic eosinophlilic pneumonia, we know many diseases in which an infiltration rich in eosinophils is found and the epidemiologic factors to be considered include exposure to certain parasites, toxic inhalation, medications and illicit drugs, as well as a history of asthma and atopy (10–12). Laboratory findings with peripheral eosinophilia occur in virtually all cases, either in the initial presentation or during the course of the disease, but are not always severe, or it can even lack since the initial clinical presentation, thereby making diagnosis difficult. In our case, at admission in the intensive care, imaging was negative as well as toxicology and laboratory finding. Peripheral eosinophilia was lacking, as pathological features associated did, like a granulomatous reaction that usually occurs in infections. This excluded a lung injury induced by pathogens or drugs. Histopathology showed the alveoli filled by proteinaceous exudates with eosinophilic granulocytes without evidence of parasitosis (no evidence of neutrophylic elements or granulomatous flogosis) or other clues for use of drugs such as amphetamine, heroin, or cocaine too (pneumocytes hyperplasia), or IgE-mediated damage (vasculitis). This confirmed an acute lung injury with hypoxia-related eosinophils. This could be due to the recent drowning, as described in literature (13, 14).

## Concluding Remarks

Our diagnosis was a death by an eosinophilic pneumonia, which developed 36 h after nearly drowning in a subject with papillary fibroelastoma. This complicance is rare but possible. The case that we are reporting is important to know pathologies that are not frequent and that can lead to death, and because the microscopic tests are often important in solving the cases that come in forensic setting. The fall in the sea was due to an accidental cardiac event and there was no crime of murder or suicide or professional misconduct by intensive care physicians.

## Author Contributions

GM, EC, CB, and MN contributed equally to the manuscript.

## Conflict of Interest Statement

The authors declare that the research was conducted in the absence of any commercial or financial relationships that could be construed as a potential conflict of interest.
